# Impact of COVID-19 on dental education- a scoping review

**DOI:** 10.1186/s12909-021-03017-8

**Published:** 2021-11-20

**Authors:** Farid Farrokhi, Simin Zahra Mohebbi, Farzaneh Farrokhi, Mohammad Reza Khami

**Affiliations:** 1 Department of Community Oral Health, School of Dentistry, Tehran University of Medical Sciences, P.O. Box 1439955934, Tehran, Iran; 2 Research Centre for Caries Prevention, Dentistry Research Institute, Tehran University of Medical Sciences, Tehran, Iran; 3 Department of Community Oral Health, School of Dentistry, Shahid Beheshti University of Medical Sciences, Tehran, Iran

**Keywords:** COVID-19, Corona virus, Dentistry, Dental education, Scoping review

## Abstract

**Background:**

A new corona virus called COVID-19 and its epidemic has affected health care systems in many ways. There have also been significant changes in dental education. The present study summarizes the findings about dental education resulting from a scoping review of COVID-19 and dentistry.

**Methods:**

First, a comprehensive search of five databases (Google Scholar, Medline through PubMed, Embase, Scopus, and Cochrane Central) was conducted with the keywords: COVID-19 and its equivalent terms, dentistry, education, oral, students, curriculum, and academics. Articles related to oral health and COVID-19 were searched. Then articles on the subject of dental education were screened and reviewed.

**Results:**

Of the 1389 articles investigating COVID-19 and dentistry, 135 articles were related to dental education and its relationship with COVID-19. The most articles in this field were from the United States, India, and Saudi Arabia. Most of the articles were cross-sectional and then review articles. Based on the review the articles were divided into two main categories of changes and concerns, and opportunities and solutions. Moreover, the following themes were extracted: teaching-learning quality and methods, study career and how students are prepared, infection control policies, theses, exams and assessments, financial and economic security, students and staff’s mental health, school’s policies and curricula, knowledge of students and staff about COVID-19.

**Conclusions:**

Dental education now faces big challenges, some of which have never been experienced before. On the other hand, the epidemic has created opportunities for dental education as well. Most of these challenges and opportunities are the same around the world, and the findings of the present study can be a good help to overcome the challenges ahead as well as a good reference to find the right questions to be answered in future studies.

**Supplementary Information:**

The online version contains supplementary material available at 10.1186/s12909-021-03017-8.

## Introduction

The emergence of the new Corona Virus called SARS-Cov-2 or COVID-19 in December 2019, and the announcement of its widespread infection as pandemic in March 11, 2020 by WHO, has prominently affected the pattern of health care systems in several aspects [1]. The risk of infection transmission in health care settings has led to substantial changes in the pattern of both delivery and utilization of health care services [2]. While many patients refrain from seeking necessary health care due to fear of being infected [3], health care system has focused on essential preparation to minimize the risk of infection transmission [4].

Despite some other health care services, most dental services can be delivered only through close contact with patients. Since it is not possible for the patients to wear a mask during dental treatment, the whole responsibility of protection relies on the shoulder of oral health professionals [5]. Thus, dentistry has been affected wildly by the pandemic in its all aspects. Among these changes, the use of tele-dentistry (TD) can be mentioned as one of the suggestions. TD is a branch in which the internet and communication technology are used to remotely exchange patient information between specialists [6, 7].

Experts have identified both strengths and challenges for TD. Benefits such as saving time, reducing costs, the possibility of consulting with colleagues, easier decision making to refer patients and the possibility of using it in primary health care system are among its strengths. On the other hand, low motivation, limited use in the dental emergency, reluctance of some specialists for the use of technology and electronic tools, and the lack of appropriate infrastructure can be considered as some of the most challenging items of tele-dentistry [8, 9].

Significant changes have also occurred in dental education. The most popular change in the education in many disciplines has been restriction of education to virtual teaching and learning. Virtual learning could be in the form of online theory and practical classes and assessments. Various methods could be used in this area, such as PowerPoint presentation, live or recorded lectures, video-based or case-based learning, inter-active learning, online whiteboard teaching, and virtual models [10]. However, many of these methods are of limited use in dental education because of its practical and skill-based nature [11].

It is true that vaccination has been done in different countries, but students from many colleges around the world have not been vaccinated [12], and with regard to the various mutations of the virus, students and faculty members are still exposed to the risk of getting infected by the virus [13].

With regard to the large number of articles in the field of COVID-19 and dentistry, it seems necessary to have an overview of the studies conducted, the topics covered, and the details of studies conducted in this field, which will help to do further studies and to determine the correct direction of future research on this topic. Specifically, a comprehensive review of the reports and studies on dental education will facilitate knowledge and experience sharing, and will help dental education system to overcome the challenges of the pandemic as much as possible. Scoping reviews are a form of knowledge synthesis, which incorporate a range of study designs to comprehensively summarize and synthesize evidence with the aim of informing practice, programs, and policy and providing direction to future research priorities [14].

The present study summarizes the findings on the dental education resulted from a scoping review on COVID-19 and dentistry.

## Methodology

The present review, according to Arksey & O’Malley’s guideline [14], was done through five steps: identifying the research question, identifying relevant studies, study selection, charting and collating the data, and summarizing and reporting the data. In order not to miss any article, it was decided to first search for all articles on oral (dental, dentistry) health and COVID-19.

Inclusion criteria to identify relevant studies were whether the articles are in English or not and whether the articles are related to COVID- 19 and dentistry. First, Google search engine was used to refine the review question and specify other popular names for COVID-19 being used in various articles. The following names were retrieved: COVID-19, Corona virus (including novel corona virus 2019: ncov-2019, and human corona virus 2019: Hcov-2019), and SARS cov-2.

We searched for articles that were published from January 2019 to March 1, 2021. The databases in which relevant studies were searched were Google Scholar, Medline through PubMed, Embase, Scopus, and Cochrane Central. The following search inquiries were used:

(COVID-19 virus AND Dentistry) OR (COVID-19 virus AND Oral health care) OR (COVID-19 virus AND Dental health) OR (COVID-19 virus AND Oral health) OR (COVID-19 virus AND Dental emergencies) OR (COVID-19 virus AND Oral emergencies) OR (COVID-19 virus AND Oral) OR (COVID-19 virus AND Dental) OR (COVID-19 virus AND Oral manifestation) OR (COVID-19 virus AND Dental health care) OR (COVID-19 virus AND Dental services) OR (COVID-19 virus AND dental education) OR (COVID-19 virus AND dental academics) OR (COVID-19 virus AND dental students) OR (COVID-19 virus AND dental curriculum) OR (Coronavirus AND Dentistry) OR (Coronavirus AND Oral health care) OR (Coronavirus AND Dental health) OR (Coronavirus AND Oral health) OR (Coronavirus AND Dental emergencies) OR (Coronavirus AND Oral emergencies) OR (Corona virus AND Oral) OR (Coronavirus AND Oral manifestation) OR (Coronavirus AND Dental health care) OR (Coronavirus AND Dental services) OR (Coronavirus AND Dental education) OR (Coronavirus AND dental academics) OR (Coronavirus AND dental students) OR (Coronavirus AND dental curriculum) OR (SARS-CoV-2 virus AND Dentistry) OR (SARS-CoV-2 virus AND Oral health care) OR (SARS-CoV-2 virus AND Dental health) OR (SARS-CoV-2 virus AND Oral health) OR (SARS-CoV-2 virus AND Dental emergencies) OR (SARS-CoV-2 virus AND Oral emergencies) OR (SARS-CoV-2 virus AND Oral) OR (SARS-CoV-2 virus AND Oral manifestation) OR (SARS-CoV-2 virus AND Dental health care) OR (SARS-CoV-2 virus AND Dental services) OR (SARS-CoV-2 virus AND dental education) OR (SARS-CoV-2 virus AND dental academics) OR (SARS-CoV-2 virus AND dental students) OR (SARS-CoV-2 virus AND dental curriculum)

In the next step, the articles focusing on dental education were screened. We screened the titles and the abstracts of the papers to exclude the irrelevant studies.

Preferred Reporting Items for Systematic reviews and Meta- Analysis (PRISMA) flow chart [15] was used to report the search results.

After selecting the studies that were in line with the objective of the study and removing duplicate articles, the data extraction process was performed and the data was entered into the table. The data presented in the table comprised: title of the article, country in which the study was conducted, date of publication, field of study, type of study, purpose of the study, number of samples participating in the study, duration of data collection, and conclusion. The process of data charting was done by a minimum of two authors. Any disagreement was discussed between the the reviewers. The selected articles were then categorized separately in to different categories using the same data from table. The data was reported narratively using the criteria set for the objectives of the study. The screening was done independently by MR. K and F. F.

As emphasized by Munn et al. [16] the researchers reviewed the titles and texts of the articles thoroughly and thematically analyzed the content.

## Result

The number of articles obtained from all data bases were 5178 (PubMed: 2186 articles, Embase: 2111 articles, Scopus: 341 articles, and Google Scholar 540 articles), which was reduced to 1532 articles by removing duplicate and unrelated articles, as well as those completely in the field of medicine.

In the next step, we removed studies that had been conducted in common fields between medicine and dentistry, such as those on the olfactory and taste changes that occur in a person after being infected with the COVID-19, tracheotomy in patients with COVID-19 in the ICU, and medications administered orally to patients with COVID-19. After this step 1389 articles remained.

**Fig. 1:**
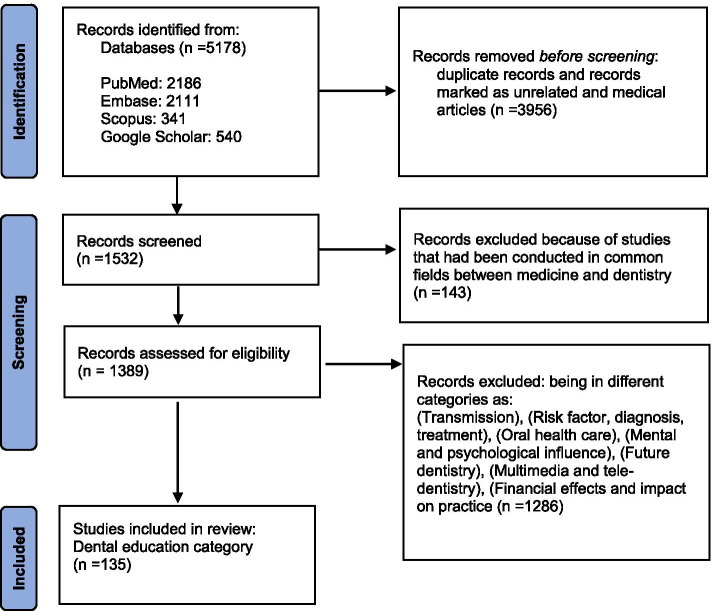
PRISMA flowchart

Out of 1389 articles obtained, 135 articles were related to dental education and its relationship with COVID-19 (Fig. 1).

This 135 articles were from different countries including: Saudi Arabia, Malta, USA, Iraq, Brazil, Turkey, UK, Italy, Canada, Taiwan, Japan, Thailand, France, India, Australia, Palestine, Pakistan, UAE, Iran, Germany, Spain, China, New Zealand, Jordan, Singapore, Netherlands, Latvia, Sweden, Hungary, Ireland, Korea, Malaysia, Nigeria, Indonesia, Egypt, Greece, Nepal and Romania.

The most active country in this field was the United States with 24 articles, followed by India and Saudi Arabia with 23 and 16 articles, respectively.

Regarding study design and article type, various formats were identified: cross sectional, review articles (critical, narrative, descriptive), letter to the editor, editorial, commentary, qualitative, report, communication (special, brief, short), perspective, interventional (before/after), technical note and case study. Around half of the articles (64) were cross sectional, followed by review articles (21).

In cross-sectional articles, the maximum number of samples was 2669, in a study entitled “COVID-19 Related Experience, Knowledge, Attitude, and Behaviors Among 2,669 Orthodontists, Orthodontic Residents, and Nurses in China: A Cross-Sectional Survey” which were conducted in China, and were collected in 3 days. The article was published on 2020/08.

The next cross-sectional article with the largest number of samples was related to a study entitled “Knowledge of dental academics about the COVID-19 pandemic: a multi-country online survey” that was from different countries (Egypt, Nigeria, Iran, Germany, Jordan, Indonesia, India, Saudi Arabia, Yemen, UK, Bosnia and Herzegovina, Italy, France, Libya, Palestine, Myanmar, Thailand, Republic of Korea, Peru, United Arab Emirates, Serbia, Kenya). The number of samples in this study was 2045. Completion of questionnaires in this study took one month and this article was published on 2020/11.

The lowest number of samples in cross-sectional studies was related to the study entitled “A nationwide survey of online teaching strategies in dental education in China” which was conducted in China on September 9, 2020, and the number of samples were 39 and it took 21 days to collect this data.

The details of all 135 articles can be found in Appendix 1[See Additional file 1].

The content and outcome of 135 articles were reviewed and these articles were divided into two main categories: 1: changes and concerns; and 2: opportunities and solutions.

## Changes and concerns imposed by COVID-19 pandemic on dental education

The articles in this category considered mainly the impact of COVID-19 pandemic and concerns raised either by experts or true surveys, on the followings:

### Teaching-learning quality and methods

One of the issues mentioned in the articles was the problems that arose in the field of teaching and the quality of learning. These issues included complications in training the students on practical skills [17, 18], concerns on quality assurance of dental education [19], online and distance education and related considerations (both theoretically and practically) [20–22]. Learning challenges due to social distance and limitations [23], and the effects that COVID-19 has had on post-graduate education [24].

The main online and distance education and related concerns included difficulties in being prepared for online education [25, 26], the criticism that most of the online courses were in the form of lectures with a little practical training content [27], lack of motivation in students in online education, poor internet connection on both students and faculty sides, and lack of necessary interaction between professors and students [28, 29].

### Future career and how students are prepared

The impact of COVID-19 on the academic resume and how students will be prepared was raised by several articles, as students’ educational activities as well as their practical skills have decreased [30–32], and students in the clinic and pre-clinic departments believed that COVID-19 would have a negative effect on their education [33, 34].

Among the challenges that will exist in the field of students’ academic and professional future were the followings: impacts on the future of dental practice [35, 36], concerns and uncertainty about employment and stability of the dental profession in the future [37], and concerns due to professional responsibility and impact on clinical performance [23, 38].

### Infection control concerns and challenges

The reviewed articles raised the following issues regarding the challenges related to infection control:

Risk of COVID-19 infection and physical health concerns [20, 23, 24, 30–32, 38], concerns about infection control and aerosol production [35, 38], challenges related to changes in personal hygiene and social habits [39], concerns about the possibility of family members becoming infected with COVID-19 virus [30], uncertainty about adequate training in infection control and proper Personal Protective Equipment (PPE) sequencing, and feeling the need for training about COVID-19 infection [24, 34].

A negative attitude was also reported about the interest in faculty members volunteering to help the medical team during an emergency [40]. On the other hand, most of the students tended to work for patients whose COVID-19 was confirmed or suspected of having the disease [41]. Reluctant to work for a patient recovered from COVID-19 disease was more common among students in the clinical phase [34].

### Theses, exams and assessments

The main challenges in this area include: problems in working on thesis [32], anxiety about exams [30], and concerns about completing clinical and graduation requirements [38, 42].

### Financial and economic security

A number of articles have addressed student financial concerns [20], the economic impact of the pandemic [35], and financial deficiencies [17].

### Students’ and staffs’ mental health

Regarding the important issue of mental health of students and staff, articles have referred to: reactions, psychological effects and stress related to COVID-19 [32, 35, 39, 43], concerns about mental health and its importance [20, 31], mental health and stress among students [17, 21, 38, 44], and the stress related to education delivery and course management [22].

### School’s policies and curricula

In this area, the articles have discussed re-evaluating the school's policies and curricula [19, 45], and suspending classes, and postponing all patient-selected care [22] (Table 1).

**Table 1:** Changes and concerns imposed by COVID-19 pandemic on dental education (Results from 135 articles)

Categories	Number of articles
Teaching-learning quality and methods	44 articles
Students and staff’s mental health	37 articles
Infection control concerns and challenges	24 articles
Future career and how students are prepared	19 articles
School’s policies and curricula	15 articles
Theses, exams and assessments	13 articles
Financial and economic security	11 articles

## Opportunities, solutions and knowledge in dental education after facing COVID-19 challenges

A number of the articles have discussed solutions and opportunities after the COVID-19 challenges, mainly in the following areas:

### Teaching-learning quality and methods

For quality and method-related challenges in teaching and learning, the following solutions have been suggested or primarily tested: further adaptation and modification of teaching methods, investment in virtual reality and the use of portable equipment (mannequins) [18, 46–48]; distance learning and virtual training as an alternative to face-to-face classes and technology acceptance to support clinical and theoretical education [17, 19, 25, 32, 42, 49, 50]; the use of information and communication technology platforms and online education methods (online and live lectures, lectures and notes sent in the virtual learning environment, receiving notes via email and content production on the website, Instagram and YouTube) [21, 28, 30, 51, 52]; inter-institutional training programs (such as the COVID program) [53]; patients data collection and discussion without the need for their presence, virtual computerized patients (VIPs), and clinical task simulations [38, 54, 55]; easier and more regular access to e-learning resources [56]; benefitting from intelligence technology for the learning process [57]; easy-to-use virtual reality (VR) device [57–59]; access to textbooks in PDF format on the faculty website [58, 60]; and using images and videos present in the ‘3D Tooth Atlas’ [61].

In the time when all face-to-face contacts such as training programs have been reduced and dental schools stopped their face-to-face, practical and clinical training under supervision [62–64] and only emergency treatments are provided in very few cases [59], it is believed that e-learning can probably be as effective as holding classes traditionally [62, 65, 66].

### Study career and how students are prepared

The shape of the oral health workforce will change and public health education will play a role in dental education [67]. It seems that the time has come for the dental workforce to be fully integrated into primary health care, and this is only possible through the support of educational institutions and teaching to dental students [17].

### Infection control policies

For this part, items can be divided in to three parts:

#### Positive effects of COVID-19 on infection control

There is an opportunity to improve infection control in dental education to increase preparedness for future infectious diseases [38]. The disease has helped students learn more about how the disease is transmitted and the efforts being made to combat the pandemic worldwide [50].

#### Protective measures and strategies suggested or implemented

These measures and strategies include:

Protecting the health of students and staff and social distance [19, 66]; proper observance of PTT standard [42]; screening of patients by telephone interview before their referral to the dental school clinic [48, 68]; continuous monitoring of staff and students, changing clothes with clean clothes when leaving school to prevent transmission of the disease to family members [68]; face shield usage, using 70% ethyl alcohol and sodium hypochlorite to disinfect surfaces between each dental visit, using rubber-dam to reduce virus transmission, using hand tools in cases of emergency treatment [69]; preoperative anti-microbial mouth rinse [70]; booking appointments for online counseling by patients [58]; and using extra oral radiographs, creating a digital platform related to the faculty to allow sending images, remote diagnosis, reviewing the results and discussing clinical cases with students [38].

#### Knowledge, attitude and practice of students/staff about infection control

Most students have been familiar with how to prevent getting the virus [71]. There was also a positive attitude when faculty members were asked about fear of COVID-19 virus in the treatment of emergencies in patients whose body temperature was controlled and aerosol-induced therapies were avoided [40, 72].

### Theses, exams and assessments

Methods suggested or implemented in this part can be mentioned as followings:

Performing various online examination methods and organizing tests completely online [67, 73–75], using lockdown browser in tests (with the lockdown browser, students cannot search for the answer in search engines such as Google) [57], and performing theoretical tests through applications such as WebEx, Google meet, Zoom, etc. to monitor students at the time of writing [58, 76].

### Financial and economic security

It has been suggested that various schemes should be planned to prevent and offset the loss of tuition and registration fees and sponsored programs [17].

### Students and staff’s mental health

The only practical suggestion in this area has been allocating counseling sessions and support programs for students who need counseling [58, 77, 78].

### School’s policies and curricula

These are the main suggestions in this area: consideration of protocols after school reopening [20]; changes in curricula (by department, faculty, or by state law) [42, 79–81]; integration of e-learning with traditional teaching methods as a hybrid strategy (resulting in flexibility, time saving and communication improvement) [56, 63, 82, 83]; sharing online education resources between schools [28]; reviewing the schedule and clinical meetings to follow the guidelines related to social distance [22, 84]; quick but thoughtful decision making and strategic planning of leadership teams, communication between universities at the national and international levels and the use of new technologies as well as student’s projects and startups in dental schools [17, 85, 86]; preparation of colleges for some rules such as the safety of students, patients and staff of the school of dentistry, training of students and staff in the practice of infection control and the proper use of PPE [68, 87, 88]; compliance with national laws and guidelines [67]; regular meetings to continuously determine the objectives and to reclassify the content to be presented in the courses [58, 89, 90]; and using free distance learning tools for both live and recorded teaching [48];

### Knowledge of students and staff about COVID-19

Mostly the dental students have had the necessary knowledge about COVID-19, its ways of transmission [40, 41, 71, 91] and potential treatment approaches [92], and have been able to correctly define “SARS-CoV-2” as “severe acute respiratory syndrome coronavirus 2”, and have been aware of the COVID-19 virus incubation period [71, 93–95]. One of the common misconceptions has been that sneezing is a sign of illness instead of a way of transmitting the virus [96].

Almost all students have been aware that hygiene and using masks are essential to prevent the spread of the virus, and proper nutrition is effective in this regard [97, 98]. Most students were aware of the measures that should be considered for personal protection against the virus. They preferred soap for hand washing [98] and agreed with the effectiveness of using PPE [40, 41], but had little information about the appropriate dental setting suggested by the guidelines [99]. Students who assumed that gloves, masks, and other protective clothing during dental work had no effect on their protection against the COVID-19 virus were significantly more likely to be students in the clinical courses [34].

It has been suggested that knowledge about the diagnostic use of Polymerase Chain Reaction (PCR) test, serological tests, and chest x-ray, as well as being familiar with treatment methods such as plasma therapy among students helps to identify and fight the virus [100–103]  (Table 2).

**Table 2:** Opportunities, solutions and Knowledge after facing challenges caused by COVID-19 on dental education (Results from 135 articles)

Categories	Number of articles
Teaching-learning quality and methods	70 articles
School’s policies and curricula	46 articles
Infection control policies	41 articles
Knowledge of students and staff about COVID-19	37 articles
Students and staff’s mental health	22 articles
Theses, exams and assessments	19 articles
Study career and how students are prepared	10 articles
Financial and economic security	2 articles

Appendix 2 shows which of the articles was discussed in each category of changes and concerns, and opportunities and solutions, separately [See Additional file 2].

## Discussion

The first reaction by many dental education institutions to the pandemic was complete shut down or restriction of education and service delivery to only emergency care [104]. When it became evident that the pandemic would probably be a long-lasting situation, the dental education institutions started to develop new and innovative strategies to continue their education, research and care delivery activities with the lowest possible infection risk for their staff, students and patients [105–107]. At the same time many of these institutions started to share their experiences and challenges in continuing their activities in the pandemic era.

In this regard, this study was conducted to investigate the effects that COVID-19 has had on dental education, as well as to review the proposed challenges and solutions and to assist in future research.

Scoping review, unlike systematic reviews, is a type of review that does not seek to answer a specific question and does not pick the best studies on the subject under study, but aims to show the perspective of the studies done [16]. Regardless of the level and method of the study, the purpose of the scoping review is to help researchers stay informed about the types of studies that have been done in a particular field.

In the first months after the beginning of the COVID-19 pandemic, most of the studies we reviewed were a kind of expert opinion in the form of letter to the editor, commentary, or review. Gradually, the number of original studies mainly in the form of cross-sectional studies have increased especially those with an online questionnaire. We found no longitudinal and prospective studies. While this was expectable because of the relatively short time passed from the onset of the pandemics, the findings of the reviewed studies should be interpreted cautiously. On the other hand, the nature of educational studies allowing short time measurement of the outcome, have enabled researchers to perform some interventional studies examining the usefulness of various educational methods such as using lockdown browser [57], comprehensive high-stakes online exam for final-year dental students [73], effect of distance education (DE) activities [108] and the Collaborative OMS Virtual Inter-Institutional Didactic (COVID) Program [53].

The negative impact of COVID-19 on the quality of education was the most important challenge raised by several reviewed studies [17, 19, 30]. This challenge has been predictable and somehow inevitable due to the necessity of social distancing and even the closure of educational institutions over a period of time. Social distance and restrictions have made e-learning much more important than ever before, as emphasized by several studies [18, 27, 51, 109–111]. This was also true for postgraduate dental education and residency programs [42, 112]. It worth to note that generally the students and teachers have reported a positive view of virtual education and believed that in a time of pandemic, virtual and online education can be used as a suitable alternative, which creates a chance that online education can be used in the future after COVID-19 pandemic [25, 113–115]. In spite of its advantages, problems with providing virtual courses do exist. The main problems in dental education, which is completely dependent on practical training, is the inability of virtual training to transfer practical skills to the level of face to face training, a point raised by several reviewed articles [20, 25, 32, 53, 116]. In a study [32], more than half of the students believed that virtual education can only partially replace face-to-face education [32], and in another study [20], students believed that practical training was damaged after the introduction of virtual education, but virtual education in other parts of the educational curriculum could be successful [20].

Given that practical training is an important part of dental education and given the advancement of technology, it seems that this problem can be solved to some extent with portable tools that students can use at home, and recording instructional videos by professors and sharing them on social media or college websites or video sharing websites like YouTube [18, 52, 117]. Portable Manikin would allow for greater modularity of classrooms hosting preclinical training and, thus, optimal use of space in faculties. Moreover, use of portable equipment would make it possible to adapt to future episodes of social distancing [18, 118].

Another issue that can be considered as a challenge in e-learning is the lack of motivation among students. In a multi-center study, most of the educational centers considered motivating students to pursue online education as one of the main problems [28]. As a response to this challenge, studies have suggested such strategies as short-term exams of chapters taught online [119] and submitting a report from the contents of the previous sessions by the students [120], and educators serving mainly as facilitators of learning [121].

Anyway, with all the challenges ahead, e-learning seems to be an integral part of the future of dental education, especially after the COVID-19 pandemic. Exposure to the Covid-19 virus may force educators and teachers to revolutionize teaching [122, 123]. Researchers believe that new tools should be used in various aspects of education and the education system should be updated to adapt to different conditions [57, 124, 125]. It seems that education in the future after COVID-19 will be hybrid that means in-person and clinical training will be combined with virtual education [63, 126, 127].

The effects that COVID-19 has on education will have a direct impact on how students prepare and graduate, as well as on various aspects of their careers [23, 31, 34, 38]. Postgraduate students also have been concerned about completing graduation requirements and not having sufficient practical skills [42]. The pandemic has intensified concerns about job shortages and difficult employment. Students who believed that their future work schedule would change after the COVID-19 pandemic, were even more stressed [128]. These concerns were due to the possibility of limited employment [37, 128]. On the other hand, COVID-19 provides a good opportunity for students to become more familiar with the concept of pandemics and the importance of global health efforts during the COVID-19 pandemic [50], and to pay more attention to the importance of primary health care workforce role in keeping society healthy [67]. Furthermore, it seems that the time has come for a fundamental overhaul of the integration of community health and dental education. The situation is changing in such a way that public health education will play an important role in dental education in the future and also changes the shape of the oral health workforce [67].

Another aspect of pandemic-induced change in the pattern of dental practice is the need for more emphasis on infection control by the dentistry profession [35, 129, 130]. The dental schools should not only implement strict infection control policies and strategies [131, 132], but also should prepare their students to strictly follow similar strategies in their future career [35]. This, in turn, has led to advancements in infection control and preparedness for infectious diseases in the dental schools [38]. In this regard, the role of e-consultancy and tele medicine is crucial in minimizing the transmission of infection [133], and also to minimize physical contact.

Studying dentistry is costly in most countries, and financial security is a concern for both dental education institutes and students. After the start of the pandemic, due to the need for social distance and the lack of face-to-face education, the issue of costs became a challenge [134, 135]. There seem to be several ways to meet this challenge, such as more universities using virtual education to reduce costs, on the other hand reducing tuition and multi-stage payment or providing low-interest facilities to students [22, 136].

One of the important aspects that COVID-19 virus can affect, and indirectly we can see the trace in different areas of dental education, is the issue of stress and psychological effects on students and professors. Lack of progress in practical training and research, concern about getting the disease through patients, worries about the family getting the virus, and worries about upcoming exams could be some of the reasons [30, 31, 137, 138]. These stresses may have adverse effects on students’ concentration, which will affect both their academic achievement and the treatment of patients, as well as their accuracy in practical work and dealing with patients. In order to maintain the health and efficiency of students, the issues that increase their concern should be reduced [139, 140], and to manage fear during illnesses such as COVID-19, dental schools should be prepared for psychological support [87, 141–143]. Students may need counseling and psychological support during and after the COVID-19 pandemic to minimize the negative impacts [21, 39, 144].

Studies on knowledge and attitudes of dental students and dental school staff towards COVID-19 and necessary preparation against its transmission have shown promising results [40, 41, 71, 96, 97]. Of course, with the changes in the guidelines and rules for infection control after the pandemic and new content that is discovered and published daily on the COVID-19 virus, it is necessary for students, faculty members, and staff to be trained and updated about the virus itself and how to deal with it [145–148]. Passing the training course in connection with covid-19 would have a direct relationship with improve in participants’ knowledge [41, 149–153].

## Conclusion

Due to COVID-19 pandemic, dental education has now faced big challenges some of which have never been experienced before. And although the vaccination is now available, several dental students around the world have not yet received it due to the global and local inequalities in COVID-19 vaccine distribution. On the other hand, the pandemic has created some opportunities for dental education as well. Most of these challenges and opportunities are the same among universities, educational institutions, faculty members and students around the world. Thus, the findings of this review can be a good help to overcome the challenges ahead and also a good reference to find the suitable questions for future studies to answer.

## Supplementary Information

**Additional file 1.** Summary of chosen articles about the impact of COVID 19 on dental education. Information (Article title, country, year, design of study, aim of study, number of participants, data collection duration, conclusion) of 135 articles which are about the impact of COVID-19 on dental education.

**Additional file 2: Table S2.** Changes and concerns imposed by COVID-19 pandemic on Dental education (Results from 135 Articles). Articles that discuss about changes and concerns imposed by COVID-19 pandemic on Dental education. **Table S3.** Opportunities, solutions and Knowledge after facing challenges caused by COVID-19 on Dental education (Results from 135 Articles). Articles that discuss about opportunities, solutions and Knowledge after facing challenges caused by COVID-19 on Dental education

## Data Availability

All data generated or analyzed during this study are included in this published article [Additional file 1].

## Abbreviations

WHO: World Health Organization
COVID-19: Coronavirus disease 2019
