# Supplementary material for: Impact of COVID-19 on dental education- a scoping review
Source: BMC Med Educ. 2021 Nov 20;21:587. doi: 10.1186/s12909-021-03017-8 (PMC8604706; doi:10.1186/s12909-021-03017-8)
Supplement: Supplementary file 1 — Additional file 1. Summary of chosen articles about the impact of COVID 19 on dental education. Information (Article title, country, year, design of study, aim of study, number of participants, data collection duration, conclusion) of 135 articles which are about the impact of COVID-19 on dental education. [file 12909_2021_3017_MOESM1_ESM.docx]

**Table 1: Summary of chosen articles about the impact of COVID 19 on dental education**

| **Number** | **Articles title** | **Country** | **Year** | **Design of study** | **Aim of study** | **Number of participants** | **Data collection duration** | **Conclusion** |
| --- | --- | --- | --- | --- | --- | --- | --- | --- |
| 1. | Awareness of, Beliefs about, Practices of, and Barriers to Tele-dentistry among Dental Students and the Implications for Saudi Arabia Vision 2030 and Coronavirus Pandemic | Saudi Arabia | 10.2020 | Cross-sectional | To investigate tele-dentistry knowledge, attitudes, and practices, and barriers to its use among dental students and teaching staff | 314 | x | Dental students seem to know little about tele-dentistry. However, they are open to learning and using it |
| 2. | A descriptive pilot study of the immediate impacts of COVID-19 on dental and dental hygiene students' readiness and wellness | US | 10.2020 | Cross-sectional | To assess the immediate impacts of COVID-19 on students’ readiness to enter clinical practice or residency and its association with well-being | 252 | 3 weeks | Academic dental institutions need to be responsive to the heightened anxiety and uncertainly levels of students and provide responsive training and support to mitigate its effects |
| 3. | Evaluation of technology-based learning by dental students during the pandemic outbreak of coronavirus disease 2019 | Iraq | 08.2020 | Cross-sectional | To evaluate different aspects of TB learning amongst undergraduate dental students during COVID-19 | 832 | x | Integrating Technology Based learning into the dental education curriculum is an essential step in enhancing the acceptance of TB learning in the future |
| 4. | Impact of COVID-19 Pandemic on Dental Education, Research, and Students | Saudi Arabia | 06.2020 | Review article | x | x | x | There is a severe underestimation of the role of the facilities and innovative technologies for e-oral health education and services, as well as tele-dentistry |
| 5. | COVID 19 pandemic unveiling the opportunities and challenges in orthodontic training | Brazil | 08.2020 | Editorial | x | x | x | Even though some aspects of teaching can and will be adequately substituted by online lecturing, treatment skills are yet to be developed and performed on patients, which is not only about technique, but understanding of patient-doctor relationship. |
| 6. | Evaluation of knowledge, attitudes, and clinical education of dental students about COVID-19 pandemic | Turkey | 08.2020 | Cross-sectional | To evaluate knowledge, attitudes, and clinical education of dental students about COVID-19 | 355 | 1 week | To be least affected by fears associated with the disease, dental faculties should be ready to provide psychological services to those in need |
| 7. | Novel Coronavirus (COVID-19) and Dentistry-A Comprehensive Review of Literature | UK | 05.2020 | Literature review | To review the available literature on  the relevant aspects of dentistry in relation to COVID-19 and to discuss potential impacts of COVID-19  outbreak on clinical dentistry, dental education and research | x | x | Optimization of cross-infection control protocols.  • Further focus on prevention and oral health promotion for the public.  • Patient empowerment and education.  • Increased role of e-consultancy and tele medicine.  • Further investment in relevant dental research field |
| 8. | COVID‐19 is a challenge for dental education—A commentary | Italy | 05.2020 | Commentary | x | x | x | The COVID-19 pandemic is likely to change dentistry and our training approach for the following years, with the need to reduce all situations potentially associated with risk of infection |
| 9. | A survey assessing the early effects of COVID-19 pandemic on oral and maxillofacial surgery training programs | US | 09.2020 | Cross-sectional | To evaluate how the COVID-19 pandemic affected oral and maxillofacial surgery (OMFS) training programs during the early phase of the pandemic | 95 | 1 month and 3 days | OMFS training programs should give more consideration to providing residents with adequate stress reduction resources to maintain their well-being and training and to minimize exposure risk during an evolving global epidemic |
| 10. | COVID‐19 pandemic: students’ perspectives on dental geriatric care and education | Canada | 07.2020 | Qualitative study | To explore how third- and fourth-year undergraduate dental students critically considered the impact of the pandemic on education and the practice of dental geriatrics | 115 | 2 month and 23 days | Students’ reflections highlighted the effects of the pandemic, not only on their education but also on frail older adults themselves and the profession of dentistry in general |
| 11. | Innovation of dental education during COVID-19 pandemic | Taiwan,  Japan,  Italy,  Thailand | 08.2020 | Expert opinion | To exchange the information and experience of  those dental educators from different countries to prepare for the future demand for dental  education during pandemic | x | x | The model  of dental education should be innovated to suit different situations and novelty intelligent  technology should be applied for future dental education |
| 12. | Continuing education in oral cancer during coronavirus disease 2019 (COVID-19) outbreak | Brazil | 04.2020 | Letter to the editor | x | x | x | We must be prepared for online continuing education, since second COVID-19 wave  is not ruled out, which may require new periods of confinement or  social distancing in the future. |
| 13. | The COVID-19 pandemic: implications for dental education | England | 06.2020 | Commentary | A commentary on  Iyer P, Aziz K, Ojcius D M.  Impact of COVID19 on dental education in the United States. J Dent  Educ 2020. DOI: 10.1002/jdd.12163. | x | x | • Dental schools should embrace technology to support clinical  and theoretical teaching  • There is an urgent need for further research into the risks of  dental aerosols, and mitigation of these risks  • Schools should make staff and student wellbeing a key priority |
| 14. | Clinical implications of the COVID-19 pandemic on dental education | US | 04.2020 | Letter to the editor | x | x | x | It is imperative that schools continue to create contingency  plans to accommodate for the lasting effects from the COVID19 pandemic. While a completely virtual dental curriculum is not necessary, some form of this adapted curriculum may need  to be retained in addition to manikin exercises to combat the  possible lag in patient flow in the aftermath of the COVID-19  pandemic |
| 15. | COVID-19: Perspective of a Dean of Dentistry | Canada | 07.2020 | Editorial | x | x | x | We should redesign our  infrastructure and manage occupational  hazards; ensure the safety of our  students, staff, and patients; adjust our  academic calendars; ensure academic  and research continuity while taking into  account the work-life balance and mental  health of our learners and staff; plan for  new programs to buffer the losses of  tuition fees, enrollment, and sponsored  programs; and recover from COVID19’s collateral damage. |
| 16. | Challenges Facing the Profession 2020: Dental hygiene education in the era of COVID-19 | US | 06.2020 | Editorial | To how dental hygiene education has responded, and what it could mean for us and for our profession in the future | x | x | Our transition to distance and online education that has opened up possibilities for many dental hygienists across the country to advance their education and careers without having to relocate to our physical campuses |
| 17. | Impact of COVID-19 on dental education: How could pre-clinical training be done at home? | France | 09.2020 | Letter to the editor | x | x | x | In addition to allowing greater flexibility for future standards of social distancing, portable manakins would allow  for greater modularity of classrooms hosting preclinical  training and, thus, optimal use of space in faculties.  We strongly believe that the use of portable equipment  would make it possible to adapt to future episodes of social  distancing |
| 18. | Fighting the COVID-19 challenge—Public health dentist's perspective | India | 08.2020 | Letter to the editor | x | x | x | While dental students continue classes through the virtual  medium, efforts can be made to help students appreciate concepts of disease transmission, pandemic, and to appreciate the  public health efforts taken worldwide to combat the pandemic.  Didactic classes can be helpful |
| 19. | Are dental schools adequately preparing dental students to face outbreaks of infectious diseases such as COVID-19? | India | 06.2020 | Guest Editorial | x | x | x | Infection control education needs to be expanded in the dental curriculum  itself and students of every dental school should be trained  adequately to protect them and prevent the infection from disseminating even before they see their first patient. |
| 20. | COVID-19 and the dental profession: professional tensions and ethical quandaries | Australia | 06.2020 | Policy report | This paper explores pertinent questions about dentistry, the dental profession and the nature of oral health that have been raised by the pandemic | x | x | More attention should be paid in dental education to learning mastery of different types of PPE |
| 21. | In an era of uncertainty: Impact of COVID-19 on dental education | US | 09.2020 | Cross-sectional | Evaluated the impact of COVID-19 on dental education and dental students’ experience | 145 | 2 weeks | The COVID-19 pandemic significantly impacted dental edu-cation. Our findings indicate that students are experiencing increased levels of stress and feel their clinical education has suffered. Most students appear comfortable with technology adaptations for didactic curriculum and favor masks, social distancing, and liberal use of sanitizers |
| 22. | Early Effects of COVID-19 on Oral and Maxillofacial Surgery Residency Training-Results From a National Survey | US | 08.2020 | Cross-sectional | To understand the effect of the COVID-19 pandemic on OMS residency training programs (OMSRTPs): 1) training and education; 2) availability and use of personal protective equipment (PPE); 3) experience with, and use of, screening and viral testing; 4) resident experience; and 5) program director (PD) experience and observations of the immediate and future effects on OMSRTPs | 174 | 1 month | Sweeping alterations to OMS clinical practice have occurred for those in OMSRTPs during the COVID-19 pandemic. Although the overall OMSRTP response has been favorable, residents' concerns regarding the ubiquitous availability of appropriate PPE, operative experience, and completion of graduation requirements requires further deliberation |
| 23. | COVID-19 outbreak, disruption of dental education, and the role of teledentistry | Saudi Arabia, Pakistan | 11.2020 | Special communication(overview) | Summarizes the potential role of tele-dentistry in continuing the dental educational process in terms of delivery of didactic components, clinical training, and patient care. | x | x | With modern updated devices and tools, tele-dentistry can be an effective way to prevent disruption of dental education and it can be utilized in continuing the dental educational process in this critical time of the COVID-19 outbreak |
| 24. | Impact of COVID-19 on dental education in the United States | US | 06.2020 | Review | Discusses the challenges that we face currently and offers some simple strategies to bridge the gaps in dental education to overcome this emergency | x | x | Prompt dental schools to re-evaluate their competency-based education, incorporate variations of distance learning permanently in their curricula, invest in haptic technology to improve psychomotor skills and also in faculty training for teaching through technology. Extramural rotations and inter-professional education should be strengthened in the curricula to enable dental students to have the ability to make an impact in the community and help during such a crisis |
| 25. | Introducing a comprehensive high-stake online exam to final-year dental students during the COVID-19 pandemic and evaluation of its effectiveness | UAE | 10.2020 | Cross-sectional | To introduce the application of a comprehensive high-stakes online exam to final-year dental students during the COVID-19 pandemic and evaluate its effectiveness | 94 | x | A newly introduced, multi-format, online high-stakes exam was implemented successfully to final-year dental students with minor technological issues and good satisfaction by students and staff alike |
| 26. | COVID-19: Finding silver linings for dental education | US, Canada | 10.2020 | Perspective | x | x | x | We must make intentional choices about how we wish to emerge: as individuals, as organizations, as a profession, and as a society. |
| 27. | Students' and lecturers' perspective on the implementation of online learning in dental education due to SARS-CoV-2 (COVID-19): a cross-sectional study | Germany | 09.2020 | Cross-sectional | To assess the students’ and lecturers’ perspectives on the implementation of online learning due to COVID-19, using a questionnaire survey | 277 | 8 days | Students’ and lecturers’ showed a predominantly positive perspective on the implementation of online learning, providing the chance to use online learning even beyond COVID-19 in the future curriculum |
| 28. | Distance learning during social seclusion by COVID-19: Improving the quality of life of undergraduate dentistry students | Brazil | 08.2020 | Cross-sectional | To assess the effect of distance education (DE) activities implemented due to social isolation, on the quality of life of undergraduate dentistry students | 230 | 48 hours | Facing social isolation never previously experienced by this generation, undergraduate dentistry students are at risk of reduced quality of life. Therefore, performing DE activities through devices with teacher‐student interaction is a key coping tool |
| 29. | The impacts of the COVID-19 pandemic on the teaching of dentistry in Brazil | Spain,  Brazil | 08.2020 | Brief communication | To report how Brazilians dental schools are leading with the new coronavirus pandemic | x | x | Students may need counseling and psychological support services during and after the COVID-19 pandemic to minimize the negative impacts on teaching and the development of their skills. |
| 30. | Hospital-based dental externship during COVID-19 pandemic: Think virtual! | US | 07.2020 | Letter to the editor | How to afford this clinical experience under a stay at home order during the current pandemic | 3 | x | This may be an opportunity for students in other dental schools, as well as other health care professions, to meet educational requirements and gain appreciation for clinical oral medicine if their institutions do not afford them similar opportunities. This will allow current students in a variety of health care professions to potentially advance and optimize clinical outcomes for their future patients |
| 31. | Dental education in the time of SARS-CoV-2 | Australia | 10.2020 | Review | To discuss the impact of the current pandemic on dental clinical education | x | x | The SARS-CoV-2 pandemic has created unique challenges for dental  schools worldwide. Dental educators have sought to identify these challenges and  find solutions appropriate to the stage of the pandemic specific to their geographical  location |
| 32. | The impact of COVID‐19 on dental education in North America—Where do we go next? | US,  Canada | 11.2020 | Commentary | We share the perspectives of faculty clinicians, residents and students in academic dental institutions in the United States and Canada | x | x | Dental academies, societies and industries have united to provide access to free continuing  education (CE) content and virtual conferences to promote professional collaboration. dental education programs have gained valuable experience in adapting and improving educational methodologies for students and trainees during this public health crisis. many institutions have been providing key resources to help students and staff affected by the  pandemic including mental health counselling, financial assistance, wellness workshops and virtual social interaction opportunities. Faculty and support staff meet with students regularly to hear their concerns |
| 33. | Impact of COVID‐19 epidemic on live online dental continuing education | China | 07.2020 | Cross-sectional | To assess live online dental continuing education during COVID-19 epidemic in China | 21 |  | The dental continuing education transferred from offline to online dramatically, and live online dental continuing education increased significantly during COVID-19 epidemic in China |
| 34. | COVID-19 and dental clinical practice: Students and clinical staff perceptions of health risks and educational impact | New Zealand,  Jordan | 09.2020 | Cross-sectional | Investigated dental students and clinical staff perceptions of health risks and impacts on clinical competence of working in teaching clinics during the COVID-19 outbreak | 76 | 1 week | The challenges of the COVID-19 pandemic also bring opportunities for dental education development; mainly in terms of infection preventionand control improvement, preparedness to respond to future contagious diseases, and exploring the benefits of online learning in dentistry |
| 35. | COVID-19 pandemic and the impact on dental education: discussing current and future perspectives | Brazil | 06.2020 | Critical Review | We discuss some favorable aspects and difficulties associated  with virtual teaching and learning, searching for available tools and techniques as well as new perspectives | x | x | It is possible to  continue teaching theoretical content, keeping  students’ unequal access to quality internet in  mind, especially those with low income. Our educational councils should be  stimulated to formulate institutional policies which  generate new options for students and professors in  this new reality |
| 36. | Redeployment of dental trainees: challenges and opportunities during the COVID-19 pandemic | UK | 09.2020 | Narrative review | We outline the transferrable skills that can be adapted and utilized during the process of redeployment in response to COVID-19. | x | x | Dental trainees require a degree of adaptability to navigate a training pathway. |
| 37. | Resident Education in the Time of a Global Pandemic: Development of the Collaborative OMS Virtual Inter-institutional Didactic (COVID) Program | US | 05.2020 | Technical note | In the present report, we  have described the Collaborative OMS Virtual Inter-institutional Didactic (COVID) Program, a multi-institutional educational curriculum developed in  response to the pandemic and aimed toward OMS resident education | x | x | The long-lasting effects of the COVID-19  pandemic on OMS residency training remain unclear. Although the COVID Program alone might not fully address the multifaceted interruption in surgical training, we believe that the program has already significantly augmented the didactic experience of residents  across the United States and will continue to do so, especially as it reaches greater numbers of residents and practitioners |
| 38. | Covid-19-related challenges in dental education: Experiences from Brazil, the USA, and Australia | Singapore,  Australia,  Brazil,  US | 07.2020 | Descriptive study | To describe and compare how three dental schools from different countries (Australia, Brazil,  and the USA) have managed experiences in dental education during the COVID-19 crisis | 3 | x | Our response to these difficulties has been a  challenging and rewarding learning experience. The way we practice dentistry and deliver dental education has changed significantly as a result of the COVID-19 pandemic, and we should share our experiences and  learn from each other. |
| 39. | Challenges in oral radiology teaching during COVID-19 pandemic | Brazil | 07.2020 | Letter to the editor | x | x | x | Teaching should be adapted to the new reality with the use of tools to make distancing learning more dynamic, interactive, and appropriate for the millennial generation.3 In addition, it is worth noting that new COVID-19 waves are not ruled out, with the possibility of further isolation and social distancing periods in the next 2 years |
| 40. | COVID‐19: The Immediate Response of European Academic Dental Institutions and Future Implications for Dental Education | UK,  Netherlands,  Latvia,  Spain,  Italy,  Sweden,  Hungary,  US,  Germany,  Ireland,  France | 11.2020 | Commentary | Given the changing COVID-19 situation and the fact that the survey is still ongoing, the authors chose to present the initial results in a commentary that will be followed later by an original paper. The aim is to get the first picture of the initial response of European dental schools to the COVID-19 crisis and determine what was informing their decisions at this stage | 153 | 10 days  (still going on) | There is thus a need for ongoing research to monitor  developments and hence ADEE will be taking this work forward in an attempt to provide some of the  answers to the above. It is important that we do not miss the opportunity to research into how these  new methods of engagement with our students and colleagues have impacted the learning  environment. |
| 41. | Psychological stress among dental students at al-iraqia university after corona virus pandemic | Iraq | 07.2020 | Descriptive study | Among undergraduate dental students at Al-Iraqia University after “Covid-19” pandemic,this study was done in order to evaluate the amount of psychological stress between them. | 305 | x | As a result of this study, there is a strong relationship between stresses during dental study (online) and “Covid-19”, also this stress differs from stage to stage, ranging from high stress at 1st stage to lowest at 2nd stage |
| 42. | Self-reported dental student stressors and experiences during the COVID-19 pandemic | Malta | 02.2021 | Cross-sectional | To compare the influence of coronavirus disease 2019 (COVID-19) pandemic on the student reported outcomes among dental students reading/studying different courses offered in the only dental school in Malta | 97 | 7 days | Students were greatly affected by the COVID-19 pandemic and this caused fear of losing their manual dexterity skills, anxiety related to its consequences on their long-term plans, and anxiety related to the examinations. Dental schools in turn should adapt rapidly and customize changes that are specific to the individual student cohort and their stage of training |
| 43. | “Break The Chain” to Embrace New Possibilities in Dental Education | India | 11.2020 | Review article | This paper will be dealing with the difficulties faced by students, faculties of the education institutions and patients, during the current pandemic | x | x | Dental educators now have the capabilities and technologies to modernize their approaches of teaching by adopting new digital concepts to amplify communication online. For a country to develop, education is the treasure path, and hence improving education is the key to development. we need to change our methods to be useful in all kind of situations. |
| 44. | E-Learning amid the COVID-19 Lockdown: Standpoint of Medical and Dental Undergraduates | Pakistan | 01.2021 | Cross-sectional | To evaluate the standpoint of medical and dental undergraduates regarding E-learning amid the Covid-19 lockdown | 283 | 1 month | Undergraduate medical and dental students from the said private medical college are well equipped and ready to shift towards online education |
| 45. | Impact of the COVID-19 pandemic on dental hygiene students in the Italian region of Emilia-Romagna | Italy | 12.2020 | Cross-sectional | To highlight the practical and emotional consequences of the emergency of COVID-19 on educational activities and in the training of students | 141 | 7 days | Students consider the dental hygienist as a risky profession, while the risk taken by patients is considered as low. Given the concern reported the students, it would be useful to address the issue of proper assessment of risk during the university training of dental hygienists |
| 46. | Are dental students aware of and knowledgeable about covid-19? A questionnaire-based investigation | Italy | 12.2020 | Cross-sectional | To evaluate the knowledge of dental students toward COVID-19 in a context of a health emergency | 72 | 10 days | Italian dental students had a good level of awareness of COVID-19. The most positive attitude of participants was toward the period of incubation, route of transmission and treatment. The COVID-19 outbreak reminded us the importance of knowledge on cross-infection control. |
| 47. | Psychological Distress during COVID-19 Lockdown among Dental Students and Practitioners in India: A Cross-Sectional Survey | India | 12.2020 | Cross-sectional | To screen for depression during the COVID-19 lockdown and the factors associated with it among dental students and practitioners in highly COVID-19 infected states of India. | 335 | 10 days | The results of this study indicate a high level of depression among a significant number of dental students and practitioners. It is important to monitor and address the mental health needs of dental students and practitioners during and after the lockdown. |
| 48. | Application of DenTeach in Remote Dentistry Teaching and Learning During the COVID-19 Pandemic: A Case Study | China,  Canada,  India | 01.2021 | Case study | We describe the available training and learning  models, present the developed DenTeach platform, and  demonstrate the feasibility of the DenTeach platform through  a case study | x | x | DenTeach can be used for training and  educating students in both clinical/laboratory and remote (i.e.,  home) settings due to its compact and portable size. This device  facilitates both fully remote and physical-distancing aware  teaching and learning in dentistry. Additionally, the DenTeach  platform can be useful when  dental schools are allowed to return to normal operations. Once  dental schools are reopened, there will be a surge in teaching,  practicing, and exams. DenTeach can be used to increase the  efficiency of the training process, thus allowing dental schools to  clear the backlog of activities faster |
| 49. | Pandemic proofing dental education | India | 02.2020 | Review | x | x | x | This adverse situation must stimulate the dental educators to prepare a COVID-proof curriculum to tackle any (including the current) pandemic situation and prepare academicians to be ready to face such untoward occurrences in future. This would entail redefining learning outcomes, improvising teaching protocols, and adapting current assessment practices. |
| 50. | COVID 19 Impact and Exploring New Avenue for Dental Education | India | 07.2020 | Review article | x | x | x | Considering our new reality, it is possible to continue  educating theoretical content, keeping students'  uneven access to quality internet, especially those  with a low-income background. Few  adequate replacements are available to dental  students today, considering pre-clinical and clinical  scenarios. dentistry will have to ensure a  greater convergence with medicine. We should reevaluate our way of delivering classes and lectures  and our curriculum with this new reality. There is a  need for further  research in this area to educate our students more  comprehensively. It should also stimulate our educational councils to formulate institutional  policies that generate new options. They must also  make significant investments in dental school, clinics  to adapt to the new bio-safety reality |
| 51. | Participation in an existing massive open online course in dentistry during the COVID-19 pandemic | US | 01.2021 | Brief communication | x | x | x | While many dental students, pre-dental  students, dentists, and colleagues across the spectrum of  careers are currently experiencing significant increases in  uncertainty, resources that can educate, provide structure,  and increase access to high-quality information will likely  continue to be sought after and add benefit |
| 52. | COVID-19 and Dental and Dental Hygiene Students' Career Plans | US | 01.2021 | Cross-sectional | To investigate whether dental and dental hygiene students’ career plans post-graduation were affected by the coronavirus disease 2019 (COVID-19) pandemic and to examine wellness and readiness for clinical practice among students who reported a change in career plans | 252 | 3 weeks | A comprehensive effort inclusive of adeptly designed clinical and curriculum experiences paired with wellness interventions and support tailored to students is needed. These measures need to support trainees across varying years in training and resilience levels to be effective for dental and dental hygiene students as they approach their future career intentions in the dental profession |
| 53. | The perceived impact of the COVID-19 pandemic on dental undergraduate students in the Italian region of Emilia-Romagna | Italy | 12.2020 | Cross-sectional | To investigate the perceived impact of the COVID‐19 pandemic on the career of dental students, the emotions and concerns following the spread of the epidemics and the restrictive measures introduced by the Italian government, along with the students' level of awareness about COVID‐19, the level of concern of being infected during daily and university activities (ie, teaching and training) and their perception of the likelihood of infection during clinical activities for dental practitioners and patients. | 399 | 2 weeks | The results of this survey could be used to train students to a correct risk assessment. Students reported experiencing concern whilst thinking of COVID‐19 and 6.5% of them showed symptoms related to high levels of anxiety. These data may guide Universities in trying to reduce students' anxiety by means of correct communication strategies |
| 54. | Effect of COVID-19 Pandemic on the Levels of Empathy among Undergraduate Dental Students in Pakistan | Pakistan | 12.2020 | Cross-sectional | To determine the effect of coronavirus disease 2019 (COVID-19) on the levels of empathy toward patients among undergraduate dental students in Pakistan. | 221 | x | This study showed an increase in mean empathy scores among the undergraduate dental students after COVID-19, indicating a significant improvement in their empathy levels during the time of COVID-19. |
| 55. | Dental Education in the Time of COVID-19 and Beyond | US | 01.2021 | Review | This article will provide an overview of the initial responses by dental education institutions to the impacts of COVID-19 and their modifications of operations implemented to reopen all phases of their educational programs in this "new normal" environment. It will also discuss potential long-term impacts on dental education based on the experiences of four dental schools | x | x | The opportunity for a student to complete part or all of the first year of the dental curriculum virtually, or from home, could lead to reduced costs associated with moving, living, and accommodations for this portion of dental school. Certainly, the Department of Education would have to make sweeping changes regarding the regulation of financial aid or Title IV funds. |
| 56. | A cross-sectional multicenter survey on the future of dental education in the era of COVID-19: Alternatives and implications | Saudi Arabia | 12.2020 | Cross-sectional | Investigated the procedures outlined by dental faculty members to maintain quality dental education in a safe bio environment and adequately control the risk of cross-infection | 212 | 1 month and 25 days | The future of dental education will have far-reaching changes in strategies and tools to cope with COVID-19 pandemic and the post-pandemic requirements of an effective, yet safe, dental learning environment. Dental colleges need to invest in infection precautions and in modern virtual education and training facilities. |
| 57. | COVID-19 Era: Challenges and solutions in dental education | Pakistan | 10.2020 | Narrative Review | Impact of COVID-19 on dental education has been discussed along with some solutions to these challenges. Key Words: COVID-19, Dental education, E-learning, Virtual/augmented reality | x | x | Virtual dental curriculum, as part of the comprehensive dental  curriculum, may need to be considered in foreseeable future. It is  evident from literature that AR/VR-based devices are making their way to dental education. While dentistry as a profession can never  be virtual, the future of dental education may be ‘blended’ with  components including both face-to-face and online components |
| 58. | Guidelines for innovation in dental education during the coronavirus disease 2019 pandemic | Taiwan,  Japan,  Australia,  Thailand,  Republic of korea,  China,  Malaysia,  Singapore | 12.2020 | Short communication | To exchange information and experiences that enhance educational offerings of greatest importance to dental education, it was deemed necessary to formulate clear COVID-19 guidelines that safely facilitate simulated training  courses/clinical skills training courses, as well as lecture courses, during  the current pandemic | x | x | Every school/institution needs to review  their curriculum to identify new and innovative ways to maintain high  educational standards. The content and criteria of education must meet the  minimum requirements of universities and national accreditation authorities. Further, dental schools/institutions must observe relevant local laws  and regulations and undertake measures necessary to ensure the safety  of all staff, students, and patients attending their school, institution, or  hospital. |
| 59. | The COVID-19 pandemic and dental residency training in Nigeria | Nigeria | 12.2020 | Cross-sectional | To assess the knowledge, perception and attitude of dental residents in Nigeria to the pandemic and its perceived impact on the dental residency training program across the country | 104 | 2 month | The dental residents had a very good knowledge of the epidemiology, diagnosis, prevention and treatment of COVID-19 and majority of them felt that it would affect their training residency program |
| 60. | COVID-19 associated stress among dental students | Saudi Arabia,  Egypt | 11.2020 | Cross-sectional | To assess levels of COVID-19 related stress and anxiety, changes in hygienic and social habits, as well as subjective worries regarding the dental profession among dental students | 258 | 2 weeks | Anxiety was higher in females with a  non-significant difference between students in different grades.  A high percentage of participating students reported changes in  their personal hygiene and social habits with subjective worries  regarding the dental profession, where one-third of them  agreed that they would change their profession if they had the  chance. Therefore, efforts should be made to minimize student  anxiety in colleges through counselling and creating a  supportive learning environment |
| 61. | Redeployment of Dental Core Trainees in the United Kingdom due to Coronavirus Disease 2019 | UK | 12.2020 | Cross-sectional | We decided to investigate the impact of COVID-19 on the redeployment of DCTs across the UK | 150 | 2 week | Many DCTs have been deployed to departments outside of their specialty and expressed some anxiety as a result. Inevitably, this has resulted in disruption to their training program and education over the last few months. The response across the United Kingdom has been understandably variable due to the differing demands of the hospital trusts within which the DCTs work |
| 62. | Awareness on Preventive Measures taken by Health Care Professionals Attending COVID-19 Patients among Dental Students | India | 12.2020 | Cross-sectional | To create awareness among dental students about the preventive measures taken by health care professionals while attending patients affected with COVID-19. | 100 | 1 month | Health care professionals play a vital role in improving access and quality health care for the population, and hence, they must be protected as well. Since dental students treat patients, they are vulnerable to infection as well, and must take proper safety measures |
| 63. | Impact of COVID-19 on dental education | India | 2020 | Review | x | x | x | The COVID 19 pandemic has caused tremendous challenges to dental academicians and  students across the globe, however it has paved a new pathway for revolutionary  modifications in dental education, research protocols, tele-dentistry, and clinical trials with  flexible approaches to solutions. The current circumstances should be taken as a suitable  moment for all the institutions to review their educational modalities, research practices,  biosafety protocols, to prepare the future generation of dentists, to face a second wave of the  COVID -19 disease and also future catastrophic events |
| 64. | A nationwide survey of online teaching strategies in dental education in China | China | 09.2020 | Cross-sectional | To (i) survey the overall implementation of online teaching in dental schools in mainland China using a nationwide evaluation feedback questionnaire; (ii) assess the dental school students’ evaluation of online education; and (iii) analyze the success and inadequacy in the practice of online dental education | 39 | 21 days | Our findings indicate the necessity and efficacy of the overall online teaching for dental education during the epidemic that can be further improved with the education model and pedagogical means to boost the informationization of dental education for future reference |
| 65. | An analysis of YouTube videos as educational resources for dental practitioners to prevent the spread of COVID-19 | Turkey | 02.2021 | Cross-sectional | To evaluate the quality of dentistry-related medical information about COVID-19 on YouTube as educational resources for dental practitioners | 55 | on 31 March 2020 between 9 AM and 6 PM | YouTube is a popular video broadcast site and can provide both relevant educational information and the spreading of misinformation. Health professionals should play a more active role with regard to educative information given on social media, especially YouTube, during global disease outbreaks |
| 66. | Nigerian undergraduate dental students' knowledge, perception, and attitude to COVID-19 and infection control practices | Nigeria | 09.2020 | Cross-sectional | To assess the knowledge, perception, and attitude of undergraduate dental students in Nigeria to the COVID‐19 pandemic and infection control practices | 102 | 1 month and 7 days | Although the clinical dental students had a positive attitude to  infection control practices against COVID-19, the overall knowledge of COVID-19 was barely adequate. Guidelines on COVID-19 from reputable health authorities should be reviewed by dental school authorities and disseminated to the students  to suit their clinical practice |
| 67. | Awareness on preventive measures in covid-19 among dental students - A questionnaire survey | India | 2020 | Cross-sectional | To assess awareness on the preventive measures in COVID-19 | 100 | x | The recent pandemic of COVID -19 has caused fear and confusion among people and there is not much awareness of drugs used to control COVID-19. People need to be more careful and follow social distancing and self-hygiene to prevent the spread of the disease |
| 68. | Knowledge and awareness about isolation and incubation of COVID 19 among dental students-A survey | India | 12.2020 | Cross-sectional | To study the Knowledge and awareness about isolation and incubation of COVID19 | 100 | x | Majority of the population possess adequate level of awareness about the pandemic outbreak COVID19’s,the need and importance of isolation and also about the incubation period of the coronavirus |
| 69. | Knowledge and awareness in maintaining good health practices during infection outbreak among dental students - A survey | India | 11.2020 | Cross-sectional | To assess the awareness in maintaining good health practices during infection outbreak among dental students | 100 | 1 month | It was evident that dental students are much aware of practices to be followed to maintain good health amidst an infection outbreak; it was also evident that both male and female students were equally aware of maintaining good health practices to fight infection. Though most of the dental students were aware of the preventive measures, the most crucial thing is to put it into practice |
| 70. | Awareness and knowledge of undergraduate dental students about the signs and symptoms of Corona viral infection (COVID-19), and the required infection control measures to prevent its spread | Egypt | 02.2021 | Cross-sectional | To assess the awareness and knowledge of dental students about the signs and symptoms of Corona viral infection (COVID-19) and to evaluate their awareness about the required infection control measures that should be performed during the dental treatment to control the spread of the disease | 1555 | 15 days | Dental students and interns in Cairo, Egypt, have good knowledge and awareness about COVID-19 and the necessary precautions required to provide adequate dental treatment for the patients during the pandemic COVID-19; however, the importance of infection control should be highlighted for both clinical and preclinical dental students, to provide safe dental treatment to the patients as well as protection of the dentists and healthcare workers |
| 71. | Knowledge of dentists, dental auxiliaries, and students regarding the COVID-19 pandemic in Saudi Arabia: a cross-sectional survey | Saudi Arabia | 12.2020 | Cross-sectional | To assess the knowledge of dental professionals in Saudi Arabia regarding severe  acute respiratory syndrome coronavirus 2 (SARS-CoV-2) and coronavirus disease 2019 (COVID-19 | 1033 | 1 month | Dental professionals seem to be consistent regarding their knowledge of the incubation period of  SARS-CoV-2. However, knowledge of viral survivability and recommended hand-soap washing time was significantly variable among the professionals. A high degree of apprehension toward suspected COVID-19 patients existed  among all dental professionals. Pandemic-awareness campaigns are essential among healthcare providers |
| 72. | Knowledge of Iranian dentists, dental specialists, and dental students towards covid-19: A preliminary survey of 778 subjects | Iran | 08.2020 | Cross-sectional | Assessed the knowledge of dentists, dental students, and dental specialists towards COVID-19 | 778 | 6 days | Knowledge of dentists, dental students, and specialists towards SARS-CoV-2 is generally good, except in the case of more technical questions that need more education (especially regarding handwashing) |
| 73. | Awareness of diagnostic tests for COVID among dental students | India | 2021 | Cross-sectional | To assess the awareness of diagnostic  tests for COVID-19 among dental students | 100 | x | The study concluded the respondents had moderate awareness about the diagnostic tests for  COVID-19. The COVID-19 pandemic poses a major global challenge. With a subtle clinical  presentation and asymptomatic carriage, and in the absence of specific treatment and vaccines, it  is clear that an early and accurate diagnosis is crucial for the control of the disease |
| 74. | Awareness of COVID-19 among dental students in Chennai, India- A questionnaire based study | India | 2020 | Cross-sectional | To assess the knowledge, awareness and hygiene practices regarding COVID-19 among dental students in Chennai, India during these critical times | 198 | 1 month | Most the participants seemed to have inadequate knowledge about COVID19 and precautionary measures taken to prevent COVID19 from spreading. These show vulnerabilities in the knowledge of dental students to deal with the current scenario and thus, there is a need to improve the knowledge of dental students through health education and training programs. |
| 75. | Preparing dental schools to refunction safely during the COVID-19 pandemic: an infection prevention and control perspective | Greece | 01.2021 | Review | To provide an evidence-based overview of infection prevention and control of COVID-19 for the safe operation of dental schools, having as priority to protect the students, patients, and personnel | x | 1 day | COVID-19 is an emerging, easily transmitted disease through respiratory droplets and direct contact. Dental schools should protect their students, patients, and personnel while providing essential educational and dental healthcare services. |
| 76. | Awareness of symptomatic differences COVID-19, Sars, swine flu, common cold among dental students | India | 10.2020 | Cross-sectional | To assess the awareness of symptomatic differences between viral diseases like COVID-19, SARS, Swine flu and common cold among dental students that support the prevention of emergence or re-emergence. | 100 | x | The dental students predominantly have convincing knowledge about the symptomatic differences between viral diseases like COVID-19, SARS, Swine Flu, Common cold which shows that they are competent enough to identify patients with these illnesses and thus protect themselves and the patients from exposure. |
| 77. | Dental faculty’s knowledge and attitude regarding Covid 19 disease in Qassim university, Saudi Arabia | Saudi Arabia | 10.2020 | Cross-sectional | To explore the knowledge and attitude of dental faculty at Qassim University, regarding novel COVID 19 disease. | 55 | 16 days | Qassim University dentists were mindful of the COVID-19 symptoms, means of transmission, Cross-infection control and operative protocols practiced within dental clinics. However, dentists exhibited inadequate knowledge about the specific dental procedures that safeguard the dental staff and patients from COVID-19 in context of current outbreak |
| 78. | Awareness toward COVID-19 precautions among different levels of dental students in King Saud university, Riyadh, Saudi Arabia | Saudi Arabia | 09.2020 | Cross-sectional | To determine the level of awareness among the dental students regarding COVID-19. | 412 | 3 weeks | Despite attaining fair scores on knowledge and attitudes, the low practice scores highlight the need for urgent strategies to prevent infection among dental students, including mandatory crash courses and hands-on protection measures. |
| 79. | Clinical dental students’ knowledge regarding proper dental settings for treating patient during COVID-19: A cross-sectional study | Saudi Arabia | 03.2021 | Cross-sectional | To investigate the clinical dental students’ knowledge on the proper dental setting during COVID-19 | 267 | x | It was noted that clinical dental students have low knowledge on the proper dental settings during COVID-19 pandemic that was recommended by the Saudi CDC guidelines and they must be equipped with adequate knowledge from reliable sources to overcome their insufficiencies such as a well-structured and dynamic curriculum |
| 80. | Knowledge and Awareness of COVID-19 among Dental Students, Interns, Clinicians and Academics | Saudi Arabia | 07.2020 | Cross-sectional | To evaluate and assess knowledge with regards to COVID-19 among dental students, interns, clinicians and academics in Riyadh, Saudi Arabia. | 366 |  | This study identified areas where participants lacked the knowledge in managing COVID-19 with the best methods available especially at an undergraduate level. In order to have a more efficient and appropriate response for future pandemics, dental curriculums and educational activities should pro-actively provide training opportunities |
| 81. | Behavior change due to COVID-19 among dental academics-The theory of planned behavior: Stresses, worries, training, and pandemic severity | Multiple countries | 09.2020 | Cross-sectional | The psychological impact of COVID-19 on dental academics globally and on changes in their behaviors | 1862 | 3 month | Fears and stresses were associated with greater adoption of preventive measures against the pandemic |
| 82. | General awareness about covid-19 and the role of convalescent plasma therapy in the treatment of covid-19 among post graduate dental students-a kap survey | India | 08.2020 | Cross-sectional | Assessing the general awareness about Covid-19 and knowledge about the role of Convalescent plasma therapy for treatment of COVID-19 among post graduate dental students. | 103 | x | Postgraduate dental students have a varied knowledge of COVID-19, and most of them suggested that plasma therapy is beneficial. Despite the spread and severity of COVID-19, most of them are willing to treat patients by providing palliative treatment by limiting the spread from dental clinics or hospitals |
| 83. | Academic Dental Activities and COVID-19 Pandemic | Italy, Saudi Arabia | 06.2020 | Editorial | x | x | x | In the coming months (recovery phase), recommendations and rules will be obviously updated or modified in different countries  (depending on the variable local risk of infection), in an attempt to recover from an academic lockdown of 2–3 or even more months.  It will be interesting at the start of the new academic year to  understand how dental educators evaluated academic activities |
| 84. | COVID-19: Perspectives for the management of dental care and education | Brazil | 09.2020 | Review | To list the challenges and perspectives in managing dental care in services and schools. | x | x | Dentists should seek specific and particular regulations for dental practice established by their state or country. Biosafety checklists are strongly recommended for appointments at dental services and face-to-face activities in dental schools |
| 85. | COVID-19 Related Experience, Knowledge, Attitude, and Behaviors Among 2,669 Orthodontists, Orthodontic Residents, and Nurses in China: A Cross-Sectional Survey | China | 08.2020 | Cross-sectional | To assess the current COVID-19 related experiences, knowledge, attitudes, and behaviors among orthodontists, orthodontic residents, and orthodontic nurses in China, and to identify factors associated with their self-perceived and actual level of knowledge, as well as their willingness to treat/care for COVID-19 patients | 2669 | 3 days | Before work resumption, COVID-19-related training programs are essential for the improvement of knowledge, confidence, and preparedness of orthodontic professionals. Sufficient and proper protection should also be provided to ensure safety and reduce the psychological burden on them |
| 86. | Perceived impact of the COVID-19 pandemic on orthodontic practice by orthodontists and orthodontic residents in Nigeria | Nigeria | 08.2020 | Cross-sectional | To assess the impacts of the pandemic on orthodontists and orthodontic residents in Nigeria | 98 | 1 month | Almost all respondents reported that they would change their future practice of orthodontics, particularly with respect to placing a greater emphasis on infection control. Most of the respondents reported perceived economic, psychosocial, and social impacts due to the pandemic |
| 87. | Knowledge and awareness of COVID-19 symptoms among dental students-a questionnaire based survey | India | 09.2020 | Cross-sectional | To assess the knowledge and awareness among dental professionals towards COVID-19 and reduce the spread among patients and co-workers. | 100 | x | The results of the survey showed that some knowledge gaps exist among dental students regarding this very deadly disease and hence is an urgent need for training and conduct complete aware-ness regarding this deadly disease and control it by essential precautionary methods |
| 88. | Comparison of COVID-19 Relevant Knowledge and Attitudes of Clinical and Preclinical Dental Students in Turkey | Turkey | 10.2020 | Cross-sectional | To compare the knowledge and attitudes of clinical and preclinical dental  students regarding the COVID-19 pandemic | 289 | x | More education and training courses are need in order to  improve students’ knowledge and attitudes regarding newly emerging  pandemic diseases |
| 89. | The COVID-19 post-pandemic scenario to Oral Radiology at Dental Schools | Brazil | 07.2020 | Comment | x | x | x | In the face of the post-pandemic scenario, in addition  to the recently updated biosafety guidelines for dental care, we would like to highlight some specifc recommendations and readjustments for Oral Radiology Departments at  Dental Schools. Imaging receptors should be double barriered to prevent perforation and cross contamination. The  implementation of a fully digital system is imperative, which  can minimize the risks of document-mediated transmission  from infected individuals |
| 90. | Coronavirus disease 2019 (COVID-19): Experiences and protocols from the Department of Prosthodontics at the Wuhan University | China | 07.2020 | Descriptive review | To review the experiences from the Department of Prosthodontics, Wuhan University School and Hospital of Stomatology (DP-WHUSHS), during the COVID-19 outbreak and the protocols DP-WHUSHS used to resume clinical activities after the outbreak. | x | x | Patient welfare and emergency needs should be considered amid the pandemic. Enhanced grade 2 or grade 3 PPE should be used during the outbreak. Multifactorial considerations for work resumption after the outbreak included facility preparation and management, training for employees, and clinical operation management. In-person psychological consultation and online mental wellness programs were available to employees to improve their mental wellness. Distance or online education was under rapid development to minimize the interruption in education for the students and to engage the dental community amid a pandemic |
| 91. | A perspective from a NYC chief oral and maxillofacial surgery resident during the COVID-19 pandemic | UK | 07.2020 | Letter to the editor | x | x | x | We are aware that this strategy cannot constitute a guideline, since it reflects only the situation in the sixth-largest  French city, which comprises one public university hospital and a large number of private OMFS. However, it does  reflect the importance of the partnership between public and  private professionals in providing safe and quality care to our  patients |
| 92. | Improving knowledge and assessing the awareness about COVID-19 testing among dental students-a questionnaire-based survey | India | 08.2020 | Cross-sectional | To assess the knowledge and create awareness of COVID testing among dental students | 100 | x | The awareness about covid-19 testing is the need of the hour. It requires high precision and rapid methods to enhance the efficacy of the current health system facilities in the current crisis of a pandemic |
| 93. | COVID 19 challenges in dental health care and dental schools | Romania | 04.2020 | Narrative review | x | x | x | In the new context, the dental  schools face challenges related to the need  for redesigning of the clinical space and  mixing the clinical and online dental  education, the insurance of the safety of the  dental students, teaching staff, and patients,  the adjusting of the academic calendars, the  maintenance of the academic and research  activities as well as the continuity of the oral  health care activities |
| 94. | Novel corona virus disease (COVID-19) awareness among the dental interns, dental auxiliaries and dental specialists in Saudi Arabia: A nationwide study | Saudi Arabia | 06.2020 | Cross-sectional | To investigate the current knowledge on COVID-19 among the DHCW’s; and to conduct quasi-experiment among the DHCW’s who were unaware of the disseminated COVID-19  information | 706 | x | The basic knowledge on COVID-19 among the DHCW’s in Saudi Arabia is acceptable. Timely dissemination of information by the Ministry of Health, Saudi Arabia had a positive impact  on the COVID-19 knowledge score of the DHCW’s. |
| 95. | Awareness on the treatment and vaccines for covid-19 infection among dental students-a survey | India | 07.2020 | Cross-sectional | To assess the awareness on the treatment and vaccines for COVID-19 infection among dental students | 100 | 1 month | This survey proves that most of the dental students were aware about the treatment and vaccines for the COVID 19 infection and also found that both male and female students were equally aware of the same. |
| 96. | COVID-19 and educational engagement | US | 07.2020 | Perspective | x | x | x | The ability for an OMS faculty member to measure educational engagement, particularly effective educational engagement, is essential to foster an active learning environment in residency education. |
| 97. | Strengthening health care research and academics during and after COVID19 pandemic- an Indian perspective | India | 07.2020 | perspective | Highlights the positive impact and opportunities provided by COVID-19 crisis to health care research and academic set-up. | x | x | The suggestions in the current article provide a working collaboration of students and teachers to effectively connect on virtual platforms to strengthen their research output, giving suggestions of data-sharing and rapid  review of proposals by online review ethical boards. |
| 98. | Virtual postgraduate exams and assessments: the challenges of online delivery and optimizing performance | UK | 12.2020 | Technical note | We outline the changes made and the advantages and disadvantages of these new examination formats. We discuss ways for candidates and examiners to optimize their virtual environment and develop skills that can improve performance. | x | x | x |
| 99. | The association between sleep quality, depression, anxiety and stress levels, and temporomandibular joint disorders among Turkish dental students during the  COVID-19 pandemic | Turkey | 02.2021 | Cross-sectional | To examine the association between sleep quality, depression, anxiety and stress levels, and the frequency of temporomandibular disorders in a sample of Turkish dental students during the COVID-19 pandemic | 699 | 2 month | During the COVID-19 pandemic, increased temporomandibular joint disorders were  observed with increased impaired sleep quality and higher depression, anxiety and stress levels  among dental university students |
| 100. | Impact of Lockdown on the Attitude of University Students  in South India—A Cross-Sectional Observational Study | India | 02.2021 | Cross-sectional | To assess the attitude of the university students | 1241 | 21 days | The university students were not anxious about the long-term effects of the pandemic but showed a decline in their academic performance |
| 101. | Psychological Impact of the COVID-19 Pandemic on Dental Interns in Riyadh, Saudi Arabia: A Cross-sectional Survey | Saudi Arabia | 10.2020 | Cross-sectional | To assess the psychological impact of the COVID-19 pandemic on dental interns in Riyadh, Saudi Arabia. | 110 | x | The present study found that a few percentage of the study  population were aﬀected by the outbreak of COVID-19 and have  revealed symptoms of mental health issues. |
| 102. | Virtual ARCP assessment and trainee feedback meetings:  facilitating the best experience and practice | UK | 10.2020 | Review | We present some of the important factors for optimizing the panel of virtual ARCPs and discuss methods to improve feedback given remotely for trainees. | x | x | The use of non-face-to-face meeting methods for providing  educational assessment and trainee feedback has dramatically increased during the COVID-19 pandemic. While  online platforms have some advantages over traditional face to-face meetings including convenience and reduced travel  time, they have limitations including reduced non verbal  communication and an inability to socialize and network with  panel members. |
| 103. | Perceived Preparedness of Dental Academic Institutions to Cope with the COVID-19 Pandemic: A Multi-Country Survey | Multiple countries | 02.2021 | Cross-sectional | To assess the various aspects of institutional preparedness perceived by dental academics in response to the COVID-19 pandemic in several countries around the world and to determine the institutional characteristics associated with perceived preparedness. | 1820 | 5 month | Academics from  low-income countries (LICs) and LMICs reported less availability of clinic apparel, IPC equipment,  measures before patient care, and institutional policies but more measures during patient care. There  was greater perceived preparedness in HICs and institutions |
| 104. | Applying "think-pair-share" for virtual curriculum retreat | US | 01.2021 | Technical note | x | x | x | The retreat provided an opportunity for faculty to participate in a guided dialogue via a "think-pair-share" activity that resulted in documenting the outcomes of recent curriculum changes while allowing for reflection for future improvement |
| 105. | Continuing endodontic education and COVID-19: before, during and after? | France | 07.2020 | Letter to the editor | x | x | x | Tele-technology could be a powerful tool to reach many participants in different geographical areas. It is important to have reliable means of communicating current theoretical and practical knowledge in endodontics and to establish sound methods that can help dentists to accurately assess their own performance in endodontics. |
| 106. | COVID-19 and Dental Education in Pakistan | Pakistan | 10.2020 | Short communication | x | x | x | This is the time for the dental faculty to learn how to teach online. Many online courses are available that will not only help the teaching faculty during this pandemic, but further improve their knowledge and skills. The success of e-learning depends on the attitudes and interactive teaching styles of the faculty, as well as on the experience and attitudes of students concerning technology. |
| 107. | A descriptive pilot study of the immediate impacts of COVID-19 on dental and dental hygiene students' readiness and wellness | US | 03.2021 | Descriptive pilot study | To assess the immediate impacts of COVID- 19 on students’ readiness to enter clinical practice or residency and its association with well-being (anxiety, perceived stress, coping and social support, and resilience) | 252 | 3 weeks | Academic dental institutions need to be responsive to the heightened anxiety and uncertainly levels of students and provide responsive training and support to mitigate its effects |
| 108. | An Educational Experiment Resulting from COVID-19: The Use of At-Home Waxing and Webinars for Teaching a 3-Week Intensive Course in Tooth Morphology to First Year Dental Students | US | 11.2020 | Qualitative study | To report the outcomes of presenting a 3 week intensive course in tooth morphology to first year dental students using ‘at-home’ waxing projects and webinars | 99 | 3 weeks | The overall quality of the completed waxing projects indicated ‘at-home’ waxing was  successful without direct faculty interaction by the students using series of step-by-step images and  videos present in the ‘3D Tooth Atlas’ |
| 109. | The Impact of the COVID-19 Pandemic on Online Oral Medicine Education as Viewed Through Increased Engagement in The Oral Cavity: Portal to Health and Disease | US |  | Letter to the editor | x | x | x | The Oral Cavity: Portal to Health and Disease (TOC) is a massive open online course developed by Penn Dental Medicine and distributed on the Coursera platform. |
| 110. | Knowledge and attitudes of Iranian dental students regarding infection control during the COVID-19 pandemic | Iran | 10.2020 | Cross-sectional | To measure the level of knowledge and attitude of the Iranian dental students towards COVID-19 and its infection control strategies. | 531 | x | There is a need for training sessions and additional educational programs to improve the knowledge of dental students regarding the prevention strategies for COVID-19. |
| 111. | Knowledge of dental academics about the COVID-19 pandemic: a multi-country online survey | Multiple countries | 11.2020 | Cross-sectional | Assessed COVID-19 knowledge and associated factors among dental academics in 26 countries | 2045 | 1 month | Dental academics had poorer knowledge of COVID-19 symptoms than of COVID-19 diagnostic methods. Living arrangements, academic degrees, patient load, and magnitude of the epidemic in the country were associated with COVD-19 knowledge among dental academics. Training of dental academics on COVID-19 can be designed using these findings to recruit those with the greatest need |
| 112. | Online Education Status at Dental Colleges During COVID-19 Pandemic in Nepal | Nepal | 10.2020 | Cross-sectional | To assess current practice and perception about online education among faculties of dental colleges in Nepal | 208 | 2 month | The COVID-19 pandemic has paved the way for e-learning in dental education in Nepal. Faculties agree to complete the theory syllabus and conduct sessional exams as an alternative during the lockdown period. However, they show strong reservations in carrying out practical/clinical simulations and university exams online. Training on e-learning and institutional strategy for online education is recommended |
| 113. | Perception of Dental Students of COMS-TH regarding Future of Dentistry in Nepal amid COVID-19 Pandemic | Nepal | 09.2020 | Cross-sectional | To know the perceptions of dental students of COMS-TH regarding future of dentistry in Nepal amid COVID-19 pandemic | 99 | 15 days | Most of the dental students were satisfied with dentistry as their profession and wanted National Dental Association to fix the minimum charges of each dental procedure. Also there is a need to start more post-graduation courses in existing institutions providing dental education as most of the students want to pursue it in future |
| 114. | Post COVID-19 lockdown: measures and practices for dental institutes | UAE | 10.2020 | Review | To present the currently implemented measures and propose changes in clinical dental facilities to minimize the risk of transmission | x | x | Dental practices establish a sensible workforce shift schedule, improve ventilation levels, reduce dental aerosol generating procedures, and develop a comprehensive guidance to Healthcare Workers to reduce the risk of COVID‑19 transmission |
| 115. | Practical teaching in undergraduate human and dental medical training during the COVID-19 crisis. Report on the COVID-19-related transformation of peer-based teaching in the Skills Lab using an Inverted Classroom Model | Germany | 01.2021 | Short report | Presents the adaptations needed for  this teaching concept and discusses their feasibility as well as selected evaluation results of the trial run. | 1012 | x | The majority of students found the communication of information via Moodle to be sufficient and did not experience any technical problems. An analysis of the students’ evaluation revealed a high level of overall satisfaction with the adapted teaching concept. |
| 116. | Presidential address: Quarantine guideline to protect examinees from COVID-19, clinical skill examination for Korean dental licensing examination, and computer-based testing for Korean medical, dental, and oriental medicine licensing examinations | Korea | 01.2021 | Editorial | x | x | x | The paper-and-pencil test of the Korean Medical Licensing Examination will be changed to a computer-based test (CBT) starting in 2022 |
| 117. | Rapid transition to distance learning due to COVID-19: Perceptions of postgraduate dental learners and instructors | UAE,UK, Jordan | 02.2021 | Cross-sectional | To investigate the perceptions of postgraduate dental learners and instructors about the transition to distance learning, including the changes to the learning and teaching and its efficaciousness | 71 | 1 month | The abrupt transition to distance learning, due to COVID-19, was perceived favorably by the involved stakeholders at the respective College. This unexpected change entailed overcoming plenty of challenges, but also uncovered substantial opportunities that are worth capitalizing upon in health professionals learning and teaching. |
| 118. | Retrospective investigation of organization and examination results of the state examination in restorative dentistry, endodontology and periodontology under simulated conditions in times of Covid-19 compared to standard conditions when treating patients | Germany | 12.2020 | Retrospective study  (short report) | Comparison of state examination results under simulated treatment conditions in times of Covid-19 versus patient treatment under non-pandemic conditions. | 22 | 4 hours | A simulated practical examination without patients in restorative dentistry, endodontics and periodontology resulted in matchable results compared with an examination on patients |
| 119. | Student perspective of classroom and distance learning during COVID-19 pandemic in the undergraduate dental study program Universitas Indonesia | Indonesia | 10.2020 | Cross-sectional | To evaluate the student perspective of DL compared to classroom learning (CL) in the undergraduate dentistry study program at the Faculty of Dentistry Universitas Indonesia | 301 | 1 month | Despite some challenges, dental students could adapt to the new learning methods of full DL and the majorities agreed blended learning that combined classroom and distance learning can be implemented henceforth |
| 120. | The Virtual Residency Fair: A Legacy of the COVID-19 Era and an Opportunity for the Future | US | 02.2021 | Perspectives | x | x | x | The American Association of Oral and Maxillofacial Surgeons  (AAOMS) virtual residency fair was  both timely and well-intended. This event can be greatly expanded and improved on during the coming years, having the potential to become a critical resource for future applicants, regardless of the COVID-19 pandemic. |
| 121. | Becoming a dentist from home: Online dental education during the Covid-19 pandemic | US | 11.2020 | Narrative review | x | x | x | Incorporating online learning into dental education can alter the role of the lecturer, where “educators will no longer serve mainly as distributors of content . . . but facilitators of learning and assessors of competency |
| 122. | COVID-19 associated stress among dental students | Saudi Arabia | 11.2020 | Cross-sectional | To assess levels of COVID-19 related stress and anxiety, changes in hygienic and social habits, as well as subjective worries regarding the dental profession among dental students | 258 | 2 weeks | Efforts should be made to minimize student anxiety in colleges through counselling and creating a supportive learning environment. In addition, dental schools should adequately prepare to face outbreaks of COVID-19 and other emerging infectious diseases with the latest updated information about its modes of infection |
| 123. | COVID-19: its impact on dental schools in Italy, clinical problems in endodontic therapy and general considerations | Italy | 05.2020 | Letter to the editor | x | x | x | 1.The risk for dental personnel, dental nursing staff and for dental students is high and must be managed;  2.University students are a population with a substantial number of ‘potential contacts’ that must be limited;  3.Universities in high‐risk areas should consider using online web‐based teaching; |
| 124. | Dental Core Trainees on the Intensive Care Unit - a novel training opportunity? | UK | 02.2021 | Letter to the editor | x | x | x | Redeployment of DCTs to DCC(department of critical care) is safe, and provides unique and ongoing opportunities for dental training, however, future redeployment requires COVID risk assessments for trainees prior to redeployment, and psychological support should be available throughout |
| 125. | Dental education in the covid-19 era: Challenges, solutions and opportunities | Saudi Arabia | 02.2021 | Review | Addressing aspects related to the challenges, solutions and/or opportunities in dental education during the COVID-19 era | 27 | x | The COVID-19 pandemic forced dental educators to rethink models of curricular delivery, as it disrupted traditional delivery methods. The use of technology was adapted to ensure the continuity of education. |
| 126. | The Emotional Impact of the Coronavirus Disease 2019 (COVID-19) Pandemic on the Dental Students During the Lockdown Time | Saudi Arabia | 2021 | Report | To evaluate the dental students’ perception and concerns about their training during the Coronavirus disease 2019 (COVID-19) | 364 | x | The vast majority of participants were worried about the negative impact of the disease on their clinical training. The prevalence of high levels of worry, anxiety, and stress among clinical dental students during the lockdown time emphasizes the need for psychological support activities involving university counseling services. |
| 127. | Evolution of COVID-19 Guidelines for University of Washington Oral and Maxillofacial Surgery Patient Care | US | 07.2020 | Report | Summarized the evolution of COVID-19 guidelines in 4 key areas: 1) preoperative SC2 testing; 2) PPE stewardship; 3) surgical scheduling guidelines; and 4) resident education and training for oral and maxillofacial surgery at the University of Washington, Seattle, Washington | x | x | Weekly meetings were transitioned to web-based video conferencing. Remote communication facilitated important departmental discussions and resident didactics. Resident operative exposure was decreased as a consequence of limiting operations to those deemed to urgent or emergent. The residents took advantage of this time to emphasize their didactic education through journal clubs, directed book readings, lectures, and research |
| 128. | The Impact of COVID-19 on Dental Education and Training | UK | 06.2020 | Narrative review | To explore the impact of SARS-CoV-2 on dental education and dental training programs in the UK. | x | x | COVID-19 has caused many interruptions to training. Dental core training (DCT) interviews, for example, were postponed in light of the current situation. Face to face interviews are no longer going ahead. |
| 129. | The impact of the COVID-19 pandemic on the educational process of Dental Medicine Department students in Oradea University during the lockdown period | Romania | 03.2021 | Cross-sectional | Assesses the opinions regarding the abrupt change in dental health education of students from the Department of Dentistry, Faculty of Medicine and Pharmacy, Oradea University during the lockdown period. | 163 | 4 days | The highest percentage of respondents that considered professional psychological support from university during the lockdown period useful were in the first 4 years of study. |
| 130. | Knowledge about the effects of medicinal plants against COVID-19 among dental students - A questionnaire study | India | 10.2020 | Cross-sectional | Assessing the knowledge about medicinal plants and their effect on COVID-19 in dental students to understand the effects and advantages of using medicinal plants against such diseases. | 100 | - | Most dental students are moderately aware of the effects of natural and medicinal plants against COVID-19. Natural and home  remedies are more economical and readily available to the  common man, hence the propagation of knowledge about the  various herbs and medicinal plants is essential. |
| 131. | Knowledge and attitudes among dental students about COVID-19 and its precautionary measures: A cross-sectional study | Palestine | 12.2020 | Cross-sectional | To evaluate students' knowledge and attitudes about COVID-19 and its precautionary measures. | 305 | 15 days | Dental students in this sample are adequately aware of the ongoing pandemic and its possible consequences. They feel the ethical obligation to provide safe treatment to their patients while returning to practical learning. However, fear of transmitting the infection to family and friends was a significant concern in this sample. |
| 132. | Knowledge and awareness of existing potential treatment approaches for COVID-19 among dental students | India | 10.2020 | Cross-sectional | To assess the awareness of existing potential treatment approaches for the COVID-19 outbreak among dental students | 100 | - | Most of the dental students were aware of existing potential treatment approaches for the COVID-19 outbreak. Also, the survey was useful in creating awareness and knowledge about the existing potential treatment approaches for COVID-19 among dental Students |
| 133. | Knowledge, Attitude, Behavior, and Stress Related to COVID-19 among Undergraduate Health Care Students in Jordan | Jordan | 12.2020 | Cross-sectional | To assess knowledge, attitude, and infection control measures related to the coronavirus disease 2019 (COVID-19) pandemic outbreak among Jordanian health care students | 935 | 22 days | Although the majority of students showed good knowledge scores, few of them appeared to have a serious lack of knowledge. Therefore, proper education and mentoring are necessary for students before reopening the university campuses |
| 134. | Perception of undergraduate dental students toward online lectures during covid-19 lockdown period | India | 02.2021 | Cross-sectional | To assess the perception of undergraduate dental students toward online lectures during the coronavirus disease-2019 (COVID-19) lockdown period | 356 | 1 week | Students had an overall positive attitude toward online lectures. However, students had an opinion that online lectures need further improvement to support their learning. E-learning can be considered as a useful supplemental tool for continuing dental education during this COVID-19 lockdown period |
| 135. | Views and anxiety levels of Turkish dental students during the covid-19 pandemic | Turkey | 06.2020 | Cross-sectional | To evaluate the views and anxiety levels of dental students during COVID-19 | 249 | - | Dental students in our study were anxious about COVID-19. Our results emphasize the need to create psychological support programs for dental students during and after the pandemic |
