# Supplementary material for: Impact of COVID-19 on dental education- a scoping review
Source: BMC Med Educ. 2021 Nov 20;21:587. doi: 10.1186/s12909-021-03017-8 (PMC8604706; doi:10.1186/s12909-021-03017-8)
Supplement: Supplementary file 2 — Additional file 2: Table S2. Changes and concerns imposed by COVID-19 pandemic on Dental education (Results from 135 Articles). Articles that discuss about changes and concerns imposed by COVID-19 pandemic on Dental education. Table S3. Opportunities, solutions and Knowledge after facing challenges caused by COVID-19 on Dental education (Results from 135 Articles). Articles that discuss about opportunities, solutions and Knowledge after facing challenges caused by COVID-19 on Dental education [file 12909_2021_3017_MOESM2_ESM.docx]

**Table 2: Changes and concerns imposed by COVID-19 pandemic on dental education (Results from 135 articles)**

| **Categories** | **Teaching-learning quality and methods** | **Students and staff’s mental health** | **Infection control concerns and challenges** | **Future career and how students are prepared** | **School’s policies and curricula** | **Theses, exams and assessments** | **Financial and economic security** |
| --- | --- | --- | --- | --- | --- | --- | --- |
| Article number according to the main table in additional file 1 | 1-4-5-7-8-10-11-13-15-16-17-21-23-27-28-29-30-31-33-34-38-39-43-48-49-51-52-53-56-57-59-63-64-81-83-84-93-96-100-105-106-109-119-128 | 2-4-6-7-15-21-28-29-32-34-38-41-42-43-45-47-52-53-54-59-60-61-81-84-86-90-93-99-100-101-107-122-125-126-129-133-135 | 7-9-13-14-19-21-24-31-34-42-47-53-59-60-61-63-75-77-81-85-86-88-106-123 | 5-10-19-21-22-34-42-47-49-52-53-81-86-88-93-100-106-113-129 | 4-13-15-19-20-24-31-38-55-66-84-89-93-103-106 | 5-22-25-32-34-42-48-52-53-84-98-100-128 | 5-14-15-21-32-35-38-86-93-106-125 |

**Table 3: Opportunities, solutions and Knowledge after facing challenges caused by COVID-19 on dental education (Results from 135 articles)**

| **Categories** | **Teaching-learning quality and methods** | **School’s policies and curricula** | **Infection control concerns and challenges** | **Knowledge of students and staff about COVID-19** | **Students and staff’s mental health** | **Theses, exams and assessments** | **Study career and how students are prepared** | **Financial and economic security** |
| --- | --- | --- | --- | --- | --- | --- | --- | --- |
| Article number according to the main table in additional file 1 | 1-3-4-5-7-8-9-10-11-12-13-14-15-16-17-18-19-21-22-23-26-27-28-29-30-31-32-33-34-35-37-38-39-40-42-43-44-48-49-50-51-53-56-57-63-64-65-83-84-89-90-93-96-97-102-104-105-106-108-109-112-115-117-118-119-120-121-125-128-134 | 2-4-5-8-9-10-11-13-14-15-19-21-22-24-29-32-35-37-38-40-43-44-49-50-55-56-57-58-61-64-75-80-83-84-85-89-90-93-97-102-104-114-116-124-125-127 | 5-6-8-9-10-11-13-15-18-19-20-21-22-24-26-29-31-34-38-43-46-54-56-58-59-70-75-77-78-84-85-89-90-91-93-103-106-114-116-123-127 | 1-6-18-19-21-29-46-53-59-62-66-67-68-89-70-71-72-73-74-76-77-78-79-80-82-85-87-88-92-94-95-110-111-130-131-132-133 | 2-4-9-15-29-35-40-43-47-53-59-60-81-90-101-107-122-124-125-126-129-135 | 4-7-8-11-15-25-26-32-40-43-57-63-83-93-96-98-112-116-118 | 4-15-16-26-36-40-52-55-113-129 | 4-15 |
